# Assessing past and future COVID-19 vaccine hesitancy in the United States in light of federal policy changes

**DOI:** 10.1093/haschl/qxad073

**Published:** 2023-12-01

**Authors:** Simon F Haeder

**Affiliations:** Department of Health Policy & Management, Texas A&M University, College Station, TX 77843, United States

**Keywords:** vaccination hesitancy, COVID-19, public health

## Abstract

Vaccinations provide an effective solution against the ongoing COVID-19 pandemic. Using a national survey (*n* = 3958), this study explored vaccination hesitancy for various COVID-19 vaccines and boosters, including the newly released annual vaccine for fall and winter 2023–2024. It also assessed support for federal funding for COVID-19 testing, vaccinations, and treatment. Consistent correlates of past vaccination refusal were perceptions of vaccines as safe and important, previous COVID-19 tests, concern about COVID-19, having voted for President Trump, higher religiosity, being liberal, trust in health institutions, health insurance status, and education. Other predictors showed inconsistent results across the various stages. Drivers of vaccination refusal were concerns about vaccine safety and side effects, perceived lack of information, and having previously contracted COVID-19. Intention to vaccinate was associated with concerns about COVID-19, liberalism, and trust in health institutions. Other factors were intermittently significant. We found consistent support for federal funding for those concerned about COVID-19, those concerned about the effectiveness of existing vaccines, those with trust in health institutions, those who thought vaccines are important, women, and those with lower levels of education. Opposition came from conservatives and Trump voters.

## Introduction

The COVID-19 pandemic has had tremendous influence on societies worldwide. While the most immediate health and societal effects have subsided, COVID-19 continues to pose a substantial public health threat, with repeated waves of infections year-round.^1^ Expectations for the coming winter include between 45 000 and 87 000 deaths and between 484 000 and 839 000 hospitalizations.^1^ Of course, many of the deaths and hospitalizations related to COVID-19 can be avoided with various public health measures. Achieving high rates of vaccinations, particularly with the newly released annual COVID-19 vaccine, play a crucial role in this regard. However, there are reasons to be skeptical that Americans will be eagerly seeking out vaccinations in the coming months. For one, the growing hesitancy towards vaccinations for various diseases,^2-4^ but for COVID-19 in particular, has been well documented.^5^ To make things worse, with the end of the public health emergency, COVID-19 tests, vaccines, and treatments will no longer be free of cost to all Americans.^6^ The added financial burden may further discourage uncertain individuals from moving forward with the vaccination. At the same time, millions of Americans have lost Medicaid coverage, forcing them to shoulder the burden themselves.^7^ This coincides with an environment where the general public is taking the issue of COVID-19 much less seriously, as indicated by public opinion surveys finding that a majority of Americans see the pandemic as over, and reduced adherence to public masking and testing.^8^

With the recent release of the annual COVID-19 vaccine, this study provides a unique comprehensive assessment of Americans’ vaccination behavior by bringing together assessments of past vaccine refusal with assessments of intentions to vaccinate this fall and winter. In particular, we explored the extent to which the correlates of vaccine uptake were the same or different across the various stages of the vaccination process, from the first dose to the newly released annual vaccine. Moreover, we provide a detailed examination of the reasoning behind previous vaccine refusal. The study also evaluated whether Americans would like to see federal funding for tests, vaccines, and treatments restored, an important equity and public health issue. Last, we assessed whether information about (1) recent increases in COVID-19 infection or (2) information about the costs related to COVID-19 testing, vaccinating, and treatment may affect intention to vaccinate or for support of federal funding experimentally.

## Data and methods

### Data

The data for the analyses described below were collected on August 18 and August 19, 2023. We surveyed 3958 adult Americans using Lucid. Lucid is a reputable survey company that relies on quota sampling by age, educational attainment, household income, race and ethnicity, and partisan identification. Lucid uses a double opt-in procedure. Respondents first opted into serving as a Lucid panel member. Subsequently, they agreed to participate in this survey.^9^ Compensation to Lucid was $1.50 per completed response. Lucid is frequently used to conduct survey research on US public opinion for health and social policy analysis.^7,10-12^ The data collected were closely matched to important national demographics such as race, age, sex, income, and census region. Additional details about the survey can be found in Appendix Exhibits 1-7. In order to further improve fit, we weighted the data on gender, race, income, and education based on the Current Population Survey. A total of 6657 respondents opted into the survey; 6251 (96%) consented to take the survey and 3958 respondents completed the survey (65%). Respondents were eliminated due to failure to pass 2 standard attention checks (36%) or failure to pass Captcha verification (1%). The study received approval from the institutional review boards at the appropriate institutions.

### Methodological approach

The survey produced both observational and experimental data. Experimental data from the survey experiment were analyzed using standard *t* tests accounting for survey weights. Because we conducted a number of analyses, we set a Bonferroni-adjusted *P* value of .00833 (standard *P* value: .05) for the comparisons of the various treatments. For binary outcome variables in the observational data (ie, past vaccination hesitancy), we estimated multivariate logit models with survey weights. Because logit coefficients are not directly interpretable, we utilized comparisons of predicted probabilities and average marginal effects (AMEs) to assess statistical and substantive significance.^13,14^ The correlates in the observational data for future vaccine hesitancy and support for federal funding were analyzed using ordinary least squares (OLS) regression with survey weights. The OLS approach facilitates interpretation and comparison of the results.^14^ Across, the analyses, we considered a *P* value lower than .05 as statistically significant.

### Measures

As an introduction to the survey, all respondents were notified that they would be asked several questions about COVID-19. After an initial set of questions about their experience with COVID-19 and their vaccination history, respondents were then randomly assigned to 1 of 3 groups (see Appendix Exhibit 7). Besides the control group, respondents were either assigned to a treatment that highlighted the recent increases in COVID-19 in the United States or to a treatment that informed them about the elimination of federal funding for most tests, vaccines, and treatments, as well as the expected costs to individuals for these items. After the exposure, respondents were then asked about their intention to vaccinate against COVID-19 in the future. Respondents in the control groups simply moved into questions about their intention to vaccinate this fall and winter. Afterwards, respondents were also asked whether they would like to see the federal government restore funding for COVID-19 tests, vaccines, and treatments by making them free of cost to Americans.

### Outcome measures

The analyses described below make use of several outcome measures. First, to assess past vaccination refusal,^3^ all respondents were asked which doses of the COVID-19 vaccine they received. We used this information, in conjunction with information provided by respondents about the type of vaccine they had received (Pfizer, Moderna, Novovax, Janssen [Johnson & Johnson]) to determine whether respondents were fully unvaccinated, had started or completed the initial vaccination sequence, or had received the first boosters or the second booster (see Appendix Exhibit 3). We thus created 4 dependent variables measuring whether respondents had completed (1) the first dose, (2) the initial sequence, (3) the first booster, or (4) the second booster. Each of the 4 variables is binary.

Second, we then asked those respondents who had not received 2 boosters (see Appendix Exhibit 4), “Why haven't you received all Centers for Disease Control (CDC) recommended vaccinations against COVID-19 yet?” We provided respondents with 12 distinct choices based on our reading of the extant literature, including lack of insurance coverage, lack of resources, lack of information, concerns about vaccine safety, concerns about vaccine side effects, concerns about vaccine effectiveness, whether they thought vaccines were not important, whether they saw no need for the vaccine, whether the process was too complicated, whether they lacked time, whether they already had COVID-19, and whether it was against their religious beliefs.

Third, we asked respondents about their intentions to vaccinate in the future (see Appendix Exhibit 5). Specifically, we asked those who had not received any vaccination at all whether they were “planning on getting vaccinated against COVID-19 in the near future?” For those who had been partially vaccinated, we asked them whether they were “planning on getting the vaccine booster against COVID-19 in the near future?” Last, we asked all respondents whether they were “planning on getting the new COVID-19 vaccine when it becomes available this fall?” For each question, we offered respondents a 5-point scale from “Definitely not” to “Definitely yes,” with a neutral “Might or might not” option.

Fourth, we asked all respondents whether they thought that the federal government should make available, at no cost, COVID-19 tests, vaccines, and treatments, respectively (see Appendix Exhibit 6). We again relied on the 5-point scale from “Definitely not” to “Definitely yes” with a neutral “Might or might not” option.

### Explanatory measures

To assess vaccination refusal and intention to vaccinate as well as support for federal funding, we relied on commonly used explanatory measures. First, we included various measures reflecting the experiences and behaviors of individuals related to COVID-19.^15^ Specifically, we included a measure of whether a respondent previously had COVID-19, whether they had been tested for COVID-19 before,^15^ and whether they had lost a relative or close friend to COVID-19.^16^ We expected that those who had COVID-19 would be more hesitant, while those who had been tested for COVID or lost a loved one to COVID-19 would be less hesitant. We also included a measure that assessed how concerned respondents were about COVID-19 (4-point scale)^17^ and how they assessed the risk of getting COVID-19 (5-point scale).^15^ Both should be correlated with lower levels of hesitancy. We also asked respondents how concerned they were that “current COVID-19 vaccines might not be effective against new strains of the coronavirus?” (4-point scale). We expected that those who are more concerned would be less hesitant with regard to future vaccinations. In some of our estimations, we also included binary measures for the various stages of the vaccination sequence (the initial shot, the completion of the initial sequences, and the first and second booster shot). Beyond COVID-19–related measures, we also included the canonical measures of whether respondents thought that vaccines were safe, effective, and important (all 4-point scales).^17^ For all 3, we expected that higher values would be associated with lower levels of hesitancy.

Second, politics have been shown to be a crucial factor in issues surrounding vaccination policy in general, as well as related to COVID-19 in particular.^5,16^ We thus included a dichotomous measure of whether a respondent voted for President Trump in the 2020 presidential election.^5^ We also included a binary measure for liberals (combining both “extreme liberals” and “liberals”) as well as conservatives (again combining both “extreme conservatives” and “conservatives”).^5^ We expected greater levels of hesitancy for Trump voters and conservatives, and the opposite effect for liberals. We also re-estimated all models with partisan identification as well as a more fine-grained 5-point ideological scale as a robustness check. Similarly, we queried respondents about their religiosity (4-point scale),^16^ with the expectation that more religiously active individuals would be more hesitant to get vaccinated. We also included a measure of trust in health institutions.^5^ This measure is an index (Cronbach's alpha: .870) made up of how confident that individuals are that the Centers for Disease Control and Prevention (CDC), the National Institutes of Health, the Food and Drug Administration, and medical doctors “act in the best interests of the public.” Each individual component had a 4-point scale.

Last, we included demographic information of respondents, including race and ethnicity,^5^ gender,^5^ age,^5^ income,^5^ education,^5^ and insurance status^5^ (Medicare, Medicaid, employer-sponsored insurance, uninsured). Generally, we expected that explanatory values for our assessment of support for federal funding would be analogous to those assessing hesitancy.

## Results

### Assessment of past vaccine refusal across vaccination stages

Overall, we found that 26.8% (95% CI, 25.1%-28.6%) of respondents indicated that they had not received a single dose of the COVID-19 vaccine. Moreover, we found that a cumulative 30.4% (28.6%-32.2%) of respondents had not completed the initial COVID-19 vaccination sequence. Last, 55.2% (53.1%-57.2%) of respondents had not received the first booster shot and 73.4% (71.5%-75.2%) had not received a second booster shot (see Appendix Exhibit 8). These numbers are generally in line with other reputable data sources,^18-20^ but slightly higher than CDC data.^21^

In order to assess the correlates of vaccination refusal, we estimated 4 distinct logit models for each of the COVID-19 vaccination stages described above. As logit coefficients are not directly interpretable, we also included the AMEs, the average of all the marginal effects, in cases for which their *P* value fell below .05 (Table 1). The AMEs can range from −1 to 1, with positive values indicating a higher probability and negative values indicating a reduction in probability. Several findings stand out. First, various COVID-19– and vaccine-related variables registered as strong predictors. Certain variables were consistent predictors across all stages of the vaccination sequence. Those who thought that vaccines were safe (AME: 0.075 to 0.080; *P* < .003) and those who thought that vaccines were important (0.052 to 0.080; *P* < .005) had increased probabilities of being vaccinated. Analogously, those with higher levels of concern about COVID-19 (0.032 to 0.048; *P* < .001) and those who had been tested for COVID-19 before (0.055 to 0.111; *P* < .007) also had higher levels of vaccinations. Voting for President Trump in 2020 was strongly associated with increases in vaccination refusal (−0.065 to −0.044; *P* <.029) across all stages. We found the opposite effect for liberals (0.044 to 0.061; *P* < .023) for all 4 models. There was no effect for conservatives. Uninsured individuals (−0.148 to −0.097; *P* < .015), those on Medicaid (−0.146 to −0.076; *P* < .007), and those with lower levels of education (0.032 to 0.048; *P* < .002) consistently showed lower rates of vaccinations.

**Table 1.** Correlates of past vaccine refusal by stage in the vaccination sequence.

	(1)		(2)		(3)		(4)	
	First dose completed	AME	Initial sequence completed	AME	First booster completed	AME	Second booster completed	AME
Had COVID-19	−0.132		−0.150		−0.146		−0.446^***^	−0.063
	(.304)		(.220)		(.203)		(.001)	(.001)
Concerned about COVID-19	0.347^***^	0.042	0.281^***^	0.038	0.284^***^	0.048	0.316^***^	0.045
	(.000)	(.000)	(.000)	(.000)	(.000)	(.000)	(.000)	(.000)
Perceived COVID-19 risk	0.115		0.094		−0.060		0.007	
	(.059)		(.109)		(.271)		(.902)	
Tested for COVID-19	0.761^***^	0.106	0.740^***^	0.111	0.523^***^	0.088	0.413^**^	0.055
	(.000)	(.000)	(.000)	(.000)	(.000)	(.000)	(.008)	(.006)
Lost someone to COVID-19	0.133		0.226		−0.076		0.018	
	(.330)		(.088)		(.535)		(.892)	
Vaccines are safe	0.652^***^	0.076	0.623^***^	0.080	0.459^***^	0.078	0.510^**^	0.075
	(.000)	(.000)	(.000)	(.000)	(.000)	(.000)	(.002)	(.002)
Vaccines are effective	0.071		0.081		0.206		0.290	
	(.593)		(.552)		(.126)		(.105)	
Vaccines are important	0.427^***^	0.052	0.412^***^	0.080	0.449^***^	0.076	0.535^**^	0.078
	(.000)	(.000)	(.000)	(.000)	(.001)	(.001)	(.003)	(.004)
Trust in health institutions	0.132		0.160		0.266^**^	0.045	0.360^***^	0.052
	(.142)		(.062)		(.003)	(.002)	(.000)	(.000)
Trump voter	−0.340^*^	−0.044	−0.457^***^	−0.065	−0.353^*^	−0.060	−0.356^*^	−0.049
	(.014)	(.016)	(.001)	(.001)	(.010)	(.011)	(.031)	(.028)
Liberal	0.452^**^	0.057	0.442^**^	0.061	0.342^**^	0.059	0.312^*^	0.044
	(.008)	(.005)	(.005)	(.004)	(.009)	(.011)	(.019)	(.022)
Conservative	−0.167		0.034		−0.108		0.076	
	(.249)		(.811)		(.452)		(.654)	
Religiosity	−0.086^*^		−0.094^*^	−0.013	−0.143^***^	−0.024	−0.114^**^	−0.016
	(.048)		(.025)	(.026)	(.000)	(.000)	(.009)	(.008)
Female	0.038		0.149		−0.096		−0.197	
	(.743)		(.192)		(.363)		(.083)	
Uninsured	−0.711^**^	−0.100	−0.649^**^	−0.097	−0.901^***^	−0.148	−0.953^*^	−0.117
	(.005)	(.009)	(.009)	(.014)	(.001)	(.000)	(.013)	(.003)
Medicaid	−0.557^**^	−0.076	−0.600^***^	−0.089	−0.696^***^	−0.148	−0.613^**^	−0.081
	(.004)	(.006)	(.001)	(.002)	(.000)	(.000)	(.003)	(.002)
Employer-sponsored insurance	0.381^*^	0.049	0.272		0.152		−0.123	
	(.031)	(.029)	(.104)		(.337)		(.478)	
Medicare	−0.109		−0.185		−0.094		−0.205	
	(.563)		(.323)		(.594)		(.273)	
Non-Hispanic White	−0.221		0.133		−0.030		−0.547^*^	−0.076
	(.453)		(.640)		(.920)		(.047)	(.048)
Non-Hispanic Black	−0.388		−0.031		−0.497		−1.148^***^	−0.139
	(.242)		(.924)		(.142)		(.001)	(.000)
Non-Hispanic Asian	0.950	0.106	1.281^*^	0.149	0.703		−0.271	−0.102
	(.098)	(.049)	(.015)	(.002)	(.078)		(.449)	(.008)
Hispanic	−0.366		−0.042		−0.256		−0.795^*^	
	(.271)		(.898)		(.443)		(.017)	
Income	0.133^***^	0.017	0.140^***^	0.019	0.058		0.053	
	(.001)	(.000)	(.000)	(.000)	(.106)		(.165)	
Education	0.293^***^	0.036	0.273^***^	0.037	0.285^***^	0.048	0.227^**^	0.032
	(.000)	(.000)	(.000)	(.000)	(.000)	(.000)	(.001)	(.001)
Age	−0.041		−0.044^*^		−0.049^**^		−0.025	
	(.057)		(.038)		(.009)		(.226)	
Age^2^	0.001^**^		0.001^***^		0.001^***^		0.001^**^	
	(.003)		(.001)		(.000)		(.008)	
Constant	−5.145^***^		−5.526^***^		−5.621^***^		−7.627^***^	
	(.000)		(.000)		(.000)		(.000)	
								
Observations, n	3840		3840		3840		3840	
Average prediction		0.737		0.702		0.451		0.269

Source: Authors’ survey. All models presented are logit models estimated with survey weights. *P* values in parentheses. ^***^P < .001, ^**^P < .01, ^*^P < .05.

Abbreviation: AME, average marginal effect.

Several other variables were only statistically significant at certain stages. For example, higher levels of religiosity (−0.024 to −0.013; *P* < .027) decreased the probability to vaccinate, except for the initial dose, while a previous infection with COVID-19 had no effect, except for increasing refusal for the second booster (−0.063; *P* = .001). Trust in health institutions only had an effect on the first and second booster (0.045 and 0.052; *P* < .003). Lower levels of income had these effects for the first and second dose (0.017 and 0.019; *P* < .001), while non-Hispanic Asians showed higher levels of vaccinations for the first 2 doses (0.106 and 0.149; *P* < .050).

Last, the findings were consistent in various robustness checks that included partisan identification and a 5-point scale of ideology (see Appendix Exhibit 9). In these models, Democratic partisanship was consistently associated with increased vaccination uptake, while more conservative ideology was correlated with lower rates for the initial dose as well as the first booster. We found no effects for Republicans.

### Reasons for past vaccine refusal

To better understand what drove respondents’ refusal to become fully vaccinated against COVID-19 across the 4 stages, we asked them to select from a list of 12 common concerns for vaccine hesitancy (Table 2). The list was described above. Looking at the groups of respondents in each of the different vaccination stages separately, for those without any COVID-19 vaccinations (*n* = 1157), concerns about vaccine safety (53.9%; 50.1%-57.8%), concerns about vaccine side effects (54.1%; 50.2%-57.9%), not seeing the need for a vaccine (36.6%; 32.9%-40.3%), and concerns about vaccine effectiveness (35.1%; 31.4%-38.7%) were among the most common. They were followed by a perceived lack of information (29.1%; 25.6%-32.7%) and having previously contracted COVID-19 (23.9%; 20.6%-27.2%). For those who received the initial shot but did not complete the initial series (*n* = 168), as well as those not receiving all vaccinations except for the first booster (*n* = 981), the same concerns emerged as dominant, albeit at lower levels. However, for those who had received all vaccinations except for the second booster (*n* = 706), having previously been sick with COVID-19 (30.6%; 26.1%-35.0%) and not seeing the need for the vaccine (28.8%; 24.4%-33.3%) and concerns about side effects (23.35; 19.2%-27.4%) were primary reasons for vaccine hesitancy. Cumulatively, concerns about side effects (42.1%; 39.4%-44.7%) stand out for those who had not received any booster (*n* = 2306) followed by concerns abouts vaccine safety (35.5%; 32.9%-38.1%) and not seeing the need for the vaccine (32.6%; 30.0%-35.2%) as well as the other previously mentioned concerns. The same pattern held for those not having received the second booster (*n* = 3012).

**Table 2.** Respondents’ reasons for past vaccination refusal.

	No COVID-19 vaccination			Initial sequence not completed			No first booster			No second booster			No booster cumulative			No second booster cumulative		
	Estimate	95% CI		Estimate	95% CI		Estimate	95% CI		Estimate	95% CI		Estimate	95% CI		Estimate	95% CI	
No insurance coverage	0.047	0.030	0.063	0.023	0.004	0.042	0.053	0.035	0.071	0.028	0.015	0.041	0.048	0.037	0.059	0.043	0.034	0.052
Lack of resources	0.043	0.027	0.059	0.025	0.004	0.046	0.042	0.027	0.057	0.029	0.016	0.042	0.041	0.031	0.052	0.038	0.030	0.047
Lack of information	0.291	0.256	0.327	0.182	0.113	0.251	0.143	0.113	0.172	0.107	0.076	0.138	0.217	0.195	0.240	0.190	0.171	0.209
Concerns about vaccine safety	0.539	0.501	0.578	0.198	0.110	0.286	0.178	0.145	0.210	0.075	0.048	0.102	0.355	0.329	0.381	0.285	0.264	0.307
Concerns about vaccine side effects	0.541	0.502	0.579	0.300	0.209	0.391	0.308	0.269	0.347	0.233	0.192	0.274	0.421	0.394	0.447	0.374	0.351	0.397
Concerns about vaccine effectiveness	0.351	0.314	0.387	0.185	0.107	0.263	0.143	0.114	0.173	0.074	0.049	0.098	0.247	0.223	0.270	0.204	0.185	0.223
Vaccines not important	0.150	0.122	0.178	0.074	0.028	0.120	0.070	0.049	0.091	0.027	0.013	0.042	0.109	0.092	0.126	0.089	0.075	0.102
No need for vaccine	0.366	0.329	0.403	0.323	0.225	0.422	0.284	0.245	0.322	0.288	0.244	0.333	0.326	0.300	0.352	0.317	0.294	0.339
Process too complicated	0.026	0.016	0.036	0.090	0.029	0.151	0.055	0.036	0.073	0.034	0.017	0.050	0.043	0.033	0.054	0.041	0.032	0.050
Lack of time	0.051	0.033	0.069	0.082	0.030	0.133	0.128	0.100	0.155	0.124	0.096	0.152	0.087	0.072	0.103	0.096	0.083	0.110
Already had COVID-19	0.239	0.206	0.272	0.207	0.126	0.287	0.277	0.239	0.314	0.306	0.261	0.350	0.254	0.230	0.277	0.267	0.246	0.288
Against religious beliefs	0.110	0.085	0.134	0.021	0.004	0.037	0.009	0.002	0.015	0.005	0.000	0.010	0.058	0.046	0.071	0.045	0.036	0.055
																		
Unweighted *n*	1157			168			981			706			2306			3012		

Source: Authors’ survey.

### Assessments of intentions to vaccinate

As noted above, COVID-19 has been on the rise and will likely create substantial public health impacts in the fall and winter 2023/2024. To assess whether respondents were planning on obtaining any vaccinations in the “near future,” we asked those respondents who were fully unvaccinated whether they were planning on getting vaccinated. Analogously, we asked respondents who had not received all boosters whether they were planning to receive a booster shot in the “near future.” Last, we asked all respondents about their plans about the new soon-to-be released annual COVID-19 vaccine. We note that the survey was conducted prior to the release of the new annual vaccine. However, before posing those questions to respondents, we first randomly exposed them to 1 of 2 distinct treatments focused on either the recent increases in COVID-19 or the potential costs associated with getting sick from COVID-19 with lack of federal funding. The control group, as mentioned above, did not receive any treatment. We compared respondents from either treatment with the control group for both their willingness to get vaccinated or boosted in the “near future” as well as their plans for the fall booster using *t* tests accounting for survey weights. None of the comparisons approached our predetermined levels of statistical significance. We thus pooled the data for the analyses below.

Overall, in response to our question about whether they planned to get vaccinate in the “near future,” previously unvaccinated respondents overwhelmingly indicated that they would not: 67.4% (63.9%-70.9%) said they definitely would not, while another 16.1% (13.6%-19.0%) said they probably would not (see Appendix Exhibit 10). Conversely, both positive answers received very little support: 2.5% (1.7%-3.9%) said they probably would get vaccinated, while 2.2% (1.2%-3.9%) said the definitely would. The results were not as clear for the existing booster. Here, negative answers (definitely not: 18.0% [16.0%-20.2%]; probably not: 21.2 [19.1%-23.5%]) and positive answers (probably yes: 19.3% [17.3%-21.4%]; definitely yes: 18.2% [16.2%-20.4%]) were roughly split, with another 23.3% (21.2%-25.6%) of respondents being undecided. These numbers are in line with other studies.^18^ However, intention to get vaccinated with the new annual vaccine was somewhat lower, with approximately 1 in 5 respondents indicating that they “might or might not” get vaccinated (19.0%; 17.5%-20.7%); almost 45% indicated hesitancy, with 28.4% noting that they definitely would not get vaccinated (26.5%-30.2%); and another 15.5% indicating that they would probably not get vaccinated (14.1%-17.0%). This compared to a mere 37% indicating their intention to get the booster in the fall (probably will: 16.5% [15.0%-18.0%]; definitely will: 20.7% [19.0%-22.4%]).

Our 3 distinct OLS models provide further details on predictors of intention to vaccinate (see Table 3). Concern about COVID-19 was consistently and strongly associated with higher levels of intention to vaccinate across all 3 models (0.285 to 0.323; *P* < .001). The same held for trust in health institutions (0.132 to 0.312; *P* < .003). We also found that support for President Trump in 2020 was associated with reduced levels for the current booster (−0.218; *P* = .006) as well as the future vaccine (−0.220; *P* = .000). Similarly, those who previously experienced a COVID-19 infection also showed lower levels of intention of getting vaccinated (−0.185; *P* = .008) or getting the future annual vaccine (−0.132; *P* = .003). There were consistently higher levels of intention among liberals (0.295 to 0.315; *P* < .027), but only evidence for decreased intention among conservatives for the current booster (−0.222; *P* = .010) and future annual vaccine (−0.135; *P* = .020). Moreover, those with higher trust in vaccine safety indicated that they were also more likely to seek out the current booster (0.432; *P* = .000) and new annual vaccine (0.227; *P* = .000). The same held for individuals who indicated that they were more at risk of getting COVID-19 (0.070 and 0.067; *P* < .025). We found that thinking that vaccines were effective (0.109; *P* = .036) or important (0.104; *P* = .032), respectively, were only associated with an increase in intention for the future annual vaccine. Last, receiving only a single dose of the COVID-19 vaccine was associated with increases in hesitancy for the booster (−0.222; *P* = .000) and the new annual vaccine (−0.118; *P* = .035). Those having received the vaccine but having failed to take up any boosters likewise had less intention towards seeking the current booster (−0.152; *P* = .011). However, the inverse held for the new annual vaccine (0.336; *P* = .000). Last, having received the first booster (0.404; *P* = .000) or the second booster (1.039; *P* = .000) was strongly associated with higher levels of intention towards the annual vaccine. We found no particular patterns for other variables included in the models. The findings were consistent in various robustness checks that included partisan identification and a 5-point scale of ideology (see Appendix Exhibit 11). Partisanship was associated with vaccination uptake for the previous booster as well as for the new annual COVID-19 shot, as was ideology.

**Table 3.** Correlates of future vaccine hesitancy for existing vaccine, existing booster, and future vaccine.

	(5)	(6)	(7)
	Future vaccination with existing vaccine	Future vaccination with existing booster	Future vaccination with new annual vaccine
Had COVID-19	−0.185^**^	−0.086	−0.134^**^
	(.008)	(.169)	(.003)
Concerned about COVID-19	0.285^***^	0.323^***^	0.292^***^
	(.000)	(.000)	(.000)
Perceived COVID risk	0.017	0.070^*^	0.067^**^
	(.596)	(.024)	(.001)
Tested for COVID-19	0.058	0.157	0.032
	(.380)	(.070)	(.549)
Concerned about COVID-19 vaccine effectiveness	−0.058^*^	0.023	−0.038
	(.031)	(.545)	(.097)
Lost someone to COVID-19	−0.049	0.048	0.040
	(.561)	(.467)	(.412)
First dose		−0.222^***^	−0.118^*^
		(.000)	(.035)
Initial sequence		−0.152^*^	0.336^***^
		(.011)	(.000)
First booster			0.404^***^
			(.000)
Second booster			1.039^***^
			(.000)
Trust in health institutions	0.132^**^	0.312^***^	0.246^***^
	(.002)	(.000)	(.000)
Vaccines are safe	0.078	0.432^***^	0.227^***^
	(.157)	(.000)	(.000)
Vaccines are effective	0.029	0.111	0.109^*^
	(.682)	(.184)	(.036)
Vaccines are important	0.076	0.082	0.104^*^
	(.231)	(.267)	(.032)
Trump voter	−0.104	−0.218^**^	−0.220^***^
	(.128)	(.006)	(.000)
Liberal	0.295^*^	0.301^***^	0.315^***^
	(.026)	(.000)	(.000)
Conservative	−0.056	−0.222^**^	−0.135^*^
	(.402)	(.010)	(.020)
Religiosity	0.004	−0.023	0.005
	(.842)	(.303)	(.734)
Female	−0.093	−0.084	−0.021
	(.132)	(.160)	(.617)
Uninsured	−0.060	−0.197	−0.109
	(.593)	(.202)	(.244)
Medicaid	−0.116	−0.016	−0.025
	(.247)	(.879)	(.737)
Employer-sponsored insurance	−0.166	−0.139	−0.068
	(.050)	(.114)	(.263)
Medicare	0.026	0.087	−0.011
	(.808)	(.392)	(.878)
Non-Hispanic White	−0.116	−0.208	−0.111
	(.452)	(.179)	(.266)
Non-Hispanic Black	−0.118	−0.138	−0.202
	(.538)	(.450)	(.099)
Non-Hispanic Asian	0.163	0.043	0.042
	(.561)	(.823)	(.742)
Hispanic	−0.014	−0.074	−0.054
	(.941)	(.675)	(.661)
Income	0.032	−0.021	0.004
	(.200)	(.292)	(.779)
Education	−0.013	0.079^*^	0.066^*^
	(.740)	(.030)	(.015)
Age	−0.046^***^	0.014	−0.006
	(.000)	(.152)	(.441)
Age^2^	0.000^**^	−0.000	0.000
	(.002)	(.444)	(.204)
Constant	1.736^***^	−1.169^**^	−0.525^*^
	(.000)	(.002)	(.027)
			
Observations	1060	2207	3750
*R^2^*	.320	.417	.611

All models presented are ordinary least squares (OLS) models estimated with survey weights. *P* values in parentheses. ^***^P < .001, ^**^P < .01, ^*^P < .05.

Source: Authors’ survey.

### Assessment of support for federal funding

Last, we assessed public opinion towards federal COVID-19 funding. First, we assessed whether the experimental information treatments described above had any effect. Having found no such effect, we again pooled our data. Across all 3 items, more than 70% of respondents supported federal funding to make tests (probably yes: 24.2% [22.5%-26.0%]; definitely yes: 54.7% [52.6%-56-7%]), vaccines (probably yes: 23.0% [21.3%-24.7%]; definitely yes: 56.9% [54.8%-58.9%]), and treatments (probably yes: 24.0% [22.2%-25.8%]; definitely yes: 45.7% [43.7%-47.7%]) free of cost to Americans (see Appendix Exhibit 12). Indeed, approximately 4 in 5 respondents supported making vaccines and treatments free of charge.

Estimating 3 distinct OLS models (see Table 4) to assess respondents’ support for federal payments for COVID-19 tests, vaccines, and treatments, respectively, we found consistent opposition to federal funding from conservatives across all 3 models (−0.220 to −0.132; *P* < .042). We also found opposition to federal funding for tests and vaccines for Trump voters (−0.185 and −0.140; *P* < .017), and higher levels of opposition to vaccines and treatments for those who are more religiously active (−0.052 and −0.035; *P* < .048). Consistently positive effects were present for women (0.205 to 0.238; *P* < .001), those who thought vaccines were important (0.146 to 0.176; *P* < .009), those with concerns about COVID-19 (0.108 to 0.116; *P* < .001), those with concerns about the efficacy of current vaccines against future strains (0.088 to 0.109; *P* < .002), and those with higher levels of trust in health institutions (0.127-0.224; *P* < .001). Previous vaccinations were only intermittently predictive of support. Again, the findings were consistent in various robustness checks that included partisan identification and a 5-point scale of ideology (see Appendix Exhibit 13). Ideology was a negative predictor of support while Democratic partisanship was positively aligned.

**Table 4.** Support for federal funding for COVID-19 tests, vaccines, and treatments.

	(8)	(9)	(10)
	Pay for tests	Pay for vaccines	Pay for treatments
Had COVID-19	0.045	0.027	0.009
	(.356)	(.581)	(.863)
Concerned about COVID-19	0.108^***^	0.116^***^	0.112^***^
	(.000)	(.000)	(.000)
Perceived COVID-19 risk	−0.021	−0.022	0.020
	(.363)	(.336)	(.403)
Tested for COVID-19	0.185^**^	0.080	0.111
	(.003)	(.208)	(.093)
Concerned about COVID-19 vaccine effectiveness	0.105^***^	0.088^**^	0.109^***^
	(.000)	(.001)	(.000)
Lost someone to COVID-19	0.139^**^	0.097^*^	0.120^*^
	(.003)	(.036)	(.019)
First dose	0.022	0.068	0.069
	(.669)	(.174)	(.216)
Initial sequence	0.115^*^	0.146^**^	0.054
	(.018)	(.002)	(.294)
First booster	0.000	0.012	−0.078
	(.996)	(.802)	(.180)
Second booster	0.022	0.127^**^	0.094
	(.665)	(.009)	(.097)
Vaccines are safe	−0.032	−0.042	−0.047
	(.550)	(.444)	(.406)
Vaccines are effective	0.064	0.102	0.031
	(.275)	(.097)	(.624)
Vaccines are important	0.146^**^	0.176^**^	0.150^**^
	(.008)	(.001)	(.007)
Trust in health institutions	0.200^***^	0.224^***^	0.127^***^
	(.000)	(.000)	(.001)
Trump voter	−0.185^**^	−0.140^*^	−0.073
	(.002)	(.016)	(.235)
Liberal	0.079	0.127^**^	0.143^**^
	(.080)	(.004)	(.008)
Conservative	−0.220^***^	−0.168^**^	−0.132^*^
	(.001)	(.007)	(.041)
Religiosity	−0.026	−0.052^**^	−0.035^*^
	(.123)	(.002)	(.047)
Female	0.218^***^	0.238^***^	0.205^***^
	(.000)	(.000)	(.000)
Uninsured	−0.097	−0.113	−0.086
	(.371)	(.271)	(.423)
Medicaid	0.068	−0.028	0.138
	(.366)	(.716)	(.082)
Employer-sponsored insurance	−0.022	−0.026	−0.042
	(.756)	(.707)	(.570)
Medicare	0.086	0.034	0.070
	(.231)	(.638)	(.373)
Non-Hispanic White	−0.053	−0.123	−0.194^*^
	(.568)	(.155)	(.032)
Non-Hispanic Black	0.047	−0.051	−0.076
	(.668)	(.628)	(.490)
Non-Hispanic Asian	−0.052	0.027	−0.117
	(.679)	(.818)	(.357)
Hispanic	0.003	−0.027	−0.110
	(.977)	(.791)	(.300)
Income	−0.039^**^	−0.046^***^	−0.025
	(.004)	(.001)	(.091)
Education	−0.128^***^	−0.098^***^	−0.109^***^
	(.000)	(.000)	(.000)
Age	0.016^*^	0.021^**^	0.024^**^
	(.023)	(.002)	(.002)
Age^2^	−0.000	−0.000^**^	−0.000^**^
	(.075)	(.008)	(.008)
Constant	2.495^***^	2.298^***^	2.387^***^
	(.000)	(.000)	(.000)
			
Observations	3777	3780	3780
*R^2^*	.225	.256	.156

All models presented are ordinary least squares (OLS) models estimated with survey weights. *P* values in parentheses. ^***^P < .001, ^**^P < .01, ^*^P < .05.

Source: Authors’ survey.

## Discussion and conclusion

Vaccine hesitancy emerged as a substantial public health threat before the recent COVID-19 pandemic.^4^ However, the emergence and spread of COVID-19 raised the stakes substantially because of the large and immediate societal and personal costs associated with it. Our findings here add to previous work on vaccine hesitancy and refusal related to COVID-19 by exploring differences across the various steps of the vaccination sequence.^4^ While certain predictors, like voting for President Trump and belief in vaccine safety, held across all stages, others, like perceived risk and religiosity, did not. Moreover, the study also disaggregated the underlying reasons for hesitation among those fully or partially unvaccinated. Concerns about side effects and vaccine safety were important predictors as was previous exposure to COVID-19. Moreover, the analyses here again provide important nuances across the stages of the vaccination sequence. At the same time, this study is one of the first to evaluate intention to vaccinate for the fall and winter 2023-2024, for both existing and newly released vaccines. Our findings here are in line with previous work that indicated an important role for perceptions, experience with COVID-19, as well as sociopolitical variables like voting behavior and ideology. Importantly, previous vaccinations proved to be a substantial predictor of intentions, indicating that some individuals may be forming vaccination habits among some part of the population. Our lack of findings for the experimental treatment for rising COVID-19 rates may also indicate that future increases in the incidence of the disease may not increase vaccination rates. Ultimately, findings from across all analyses indicate that high vaccination rates will be challenging to achieve with existing and future vaccines. From a broader perspective, these findings raise further concerns about spillover effects into other domains related to vaccination and public health.^22,23^

From a public health perspective, the findings raise questions about how to proceed. The need for well-designed campaigns and other public health measures is clearly evident. On the one hand, these efforts should not exclusively rely on vaccinations as the only countermeasure because realities on the ground will likely make them destined to fail unless substantial resources are mobilized. However, the prominent role that concerns about safety and vaccine side effects, lack of information, and previous exposure play in vaccine refusal provides some guidance on what issues public health campaigns should focus on. Informational campaigns should focus on utilizing trusted sources such as health care providers^3,4^ and trusted community leaders^24^ but to provide information and reduce concerns. Efforts to counter mis- and disinformation across various types of media are also crucial.^3,4^ While politically more controversial, mandates in educational settings served as effective tools to increase vaccination rates.^25,26^

Last, another crucial tool to mitigate the impact of future COVID-19 waves is federal support for testing, vaccines, and treatments, which was recently phased out. However, unlike attitudes towards vaccinations at the individual level, our findings here indicate that Americans overwhelmingly support continued federal investment. Ultimately, new federal funding could prove a crucial tool in mitigating COVID-19 in the near future. Importantly, securing this funding could prove politically beneficial with the upcoming elections in mind.

There are limitations to this study. First, commonly known limitations of cross-sectional survey research apply. Second, we relied on a (double) opt-in panel of a reputable Internet-based survey platform, which is standard today, and which is commonly used for this type of work.^9^ This company has also been used extensively to survey public opinion related to health and social policy.^7,11,12^ However, we further improved data quality by implementing 2 attention checks and a Captcha verification procedure. Moreover, our informational treatment, which showed no effect, is limited because it provides respondents with only a low intensity intervention in the form of a single factual statement. More extensive treatments may have shown additional results.

## Supplementary material

Supplementary material is available at *Health Affairs Scholar* online.

## Supplementary Material

qxad073_Supplementary_Data
