# Supplementary material for: Assessing past and future COVID-19 vaccine hesitancy in the United States in light of federal policy changes
Source: Health Aff Sch. 2023 Dec 1;1(6):qxad073. doi: 10.1093/haschl/qxad073 (PMC10986238; doi:10.1093/haschl/qxad073)
Supplement: qxad073_Supplementary_Data [file qxad073_Supplementary_Data.zip › Appendix R1.docx]

**Appendix**

**Appendix 1:** Distribution of Weights

**Appendix 2:** Comparison of Raw and Weighted Qualtrics Data to National Benchmarks

| **Variable** | **Survey Data**  **(Raw)** | **Survey Data**  **(Weighted)** | **Benchmark** | **Benchmark Source** |
| --- | --- | --- | --- | --- |
|  |  |  |  |  |
| Female | 52% | 55% | 51% | CPS |
| College Degree | 38% | 37% | 31% | CPS |
| Black | 11% | 10% | 13% | CPS |
| White | 69% | 70% | 62% | CPS |
| Hispanic | 10% | 14% | 18% | CPS |
| Democrat | 33% | 33% | 34% | ANES (Wgt.) |
| Republican | 30% | 32% | 28% | ANES (Wgt.) |
| Mean Age | 46 | 49 | 47 | ANES (Wgt.) |
| Median Income | $35 to 49,999 | $50 to $74,999 | $55 – 59,999 | ANES (Wgt.) |

Note: Comparison of the data to known population benchmarks. CPS = Current Population Survey. ANES = American National Election Study. Preference is given to CPS considering its sample size and representativeness, but make use of weighted ANES data whenever it was not possible to use CPS (i.e. CPS does not ask questions about Party ID). Weights in column two adjust for gender, education, race, age, and income. N (Survey Data) = 3,958

**Appendix 3:** Survey Questions

How concerned are you about getting COVID-19?

o Not at all

o A little

o Moderately

o Very

Compared to others, do you think your risk of getting COVID-19 is ...

o Much lower

o Somewhat lower

o About the same

o Somewhat higher

o Much higher

Have you ever been tested for COVID-19?

o Yes

o No

To your knowledge, have you had COVID-19?

o Yes

o No

Have you lost a family member or close friend due to COVID-19?

o Yes

o No

What COVID-19 vaccines have you received? Mark all that apply.

o Pfizer-BioNTech

o Moderna

o Novavax

o Janssen (Johnson & Johnson)

Which doses of the vaccine have you received? Mark all the apply.

o Initial Dose

o 2nd Dose or 3rd Dose

o 1st Booster

o 2nd Booster or 3rd Booster

How concerned, if at all, are you that current COVID-19 vaccines might not be effective against new strains of coronavirus?

o Not at all concerned

o Not too concerned

o Somewhat concerned

o Very concerned

**Appendix 4**: Reasons for COVID Vaccination Refusal

Why haven't you received all Centers for Disease Control (CDC) recommended vaccinations against COVID-19 yet? Mark all that apply.

o Do not have health insurance

o Do not have the financial resources/Too expensive

o Do not think I have enough information on vaccines

o Do not think the vaccines are safe

o Worried about the side effects of the vaccine

o Do not think the vaccines work

o Do not think the vaccines are important

o Do not think I need the vaccine

o Process is too complicated

o Do not have the time

o Already had COVID-19

o Not in line with my religious beliefs

**Appendix 5**: Intention to Vaccinate

Are you planning on getting vaccinated against COVID-19 in the near future?

o Definitely not

o Probably not

o Might or might not

o Probably yes

o Definitely yes

Are you planning on getting the vaccine booster against COVID-19 in the near future?

o Definitely not

o Probably not

o Might or might not

o Probably yes

o Definitely yes

Are you planning on getting the new COVID-19 vaccine when it becomes available this fall?

o Definitely not

o Probably not

o Might or might not

o Probably yes

o Definitely yes

**Appendix 6:** Federal Funding

Do you think the federal government should make available COVID-19 tests to Americans free of cost?

o Definitely not

o Probably not

o Might or might not

o Probably yes

o Definitely yes

Do you think the federal government should make available COVID-19 vaccines to Americans free of cost?

o Definitely not

o Probably not

o Might or might not

o Probably yes

o Definitely yes

Do you think the federal government should make available COVID-19 treatments like Paxlovid to Americans free of cost?

o Definitely not

o Probably not

o Might or might not

o Probably yes

o Definitely yes

**Appendix 7:** Treatments

Group 1: Control (no treatment). N=1,328

Group 2: Rising Covid Cases. N=1,326

In recent weeks, COVID cases have been rising in the United States as more Americans are traveling over the months.

Group 3: COVID Costs. N= 1,304

The federal funding that made COVID-19 tests, vaccines, and treatments free for Americans is coming to an end.

This means that Americans getting a test, the vaccine, or treatment will have to rely on insurance or pay out of pocket.

The expected price for each test is between $10 and $25 and it is between $80 and $130 for each vaccine.

The expected the price for each Paxlovid treatment is between $500 and $900.

**Appendix 8:** Percentage of Respondents Who Have Completed Steps in Vaccination Sequence

Source: Authors’ survey.

Notes: Confidence intervals omitted for clarity.

**Appendix 9:** Correlates of Past Vaccine Hesitancy by Stage in the Vaccination Sequence, Alternative Specification

|  | (1) | (2) | (3) | (4) | (5) | (6) | (7) | (8) |
| --- | --- | --- | --- | --- | --- | --- | --- | --- |
| VARIABLES | **First Dose** | | **Initial Sequence** | | **First Booster** | | **Second Booster** | |
|  |  |  |  |  |  |  |  |  |
| Had COVID | -0.111 | -0.106 | -0.134 | -0.132 | -0.143 | -0.143 | -0.441*** | -0.438*** |
|  | (0.398) | (0.422) | (0.278) | (0.287) | (0.218) | (0.217) | (0.001) | (0.001) |
| Concerned about COVID | 0.486*** | 0.507*** | 0.402*** | 0.416*** | 0.332*** | 0.349*** | 0.336*** | 0.336*** |
|  | (0.000) | (0.000) | (0.000) | (0.000) | (0.000) | (0.000) | (0.000) | (0.000) |
| Perceived COVID risk | 0.134* | 0.134* | 0.113 | 0.112 | -0.063 | -0.063 | 0.003 | 0.002 |
|  | (0.029) | (0.029) | (0.055) | (0.058) | (0.251) | (0.244) | (0.951) | (0.970) |
| Tested for COVID | 0.757*** | 0.755*** | 0.734*** | 0.731*** | 0.509*** | 0.507*** | 0.409** | 0.404* |
|  | (0.000) | (0.000) | (0.000) | (0.000) | (0.001) | (0.001) | (0.009) | (0.010) |
| Concerned about COVID vaccine effectiveness | -0.292*** | -0.294*** | -0.255*** | -0.257*** | -0.094 | -0.096 | -0.044 | -0.044 |
|  | (0.000) | (0.000) | (0.000) | (0.000) | (0.122) | (0.112) | (0.511) | (0.513) |
| Lost someone to COVID | 0.134 | 0.131 | 0.222 | 0.222 | -0.065 | -0.066 | 0.034 | 0.036 |
|  | (0.334) | (0.349) | (0.097) | (0.097) | (0.598) | (0.591) | (0.798) | (0.792) |
| Vaccines are safe | 0.650*** | 0.655*** | 0.613*** | 0.619*** | 0.462*** | 0.469*** | 0.505** | 0.505** |
|  | (0.000) | (0.000) | (0.000) | (0.000) | (0.000) | (0.000) | (0.002) | (0.002) |
| Vaccines are effective | 0.048 | 0.046 | 0.056 | 0.056 | 0.204 | 0.202 | 0.269 | 0.272 |
|  | (0.718) | (0.733) | (0.680) | (0.682) | (0.139) | (0.140) | (0.140) | (0.135) |
| Vaccines are important | 0.451*** | 0.459*** | 0.434*** | 0.442*** | 0.449*** | 0.460*** | 0.557** | 0.553** |
|  | (0.000) | (0.000) | (0.000) | (0.000) | (0.001) | (0.001) | (0.002) | (0.002) |
| Trust in Health Institutions | 0.128 | 0.150 | 0.154 | 0.169* | 0.264** | 0.281** | 0.336*** | 0.338*** |
|  | (0.161) | (0.097) | (0.076) | (0.049) | (0.003) | (0.002) | (0.001) | (0.001) |
| Democrat | 0.317* | 0.393* | 0.368* | 0.424** | 0.204 | 0.279* | 0.503*** | 0.498*** |
|  | (0.048) | (0.013) | (0.015) | (0.004) | (0.120) | (0.030) | (0.000) | (0.000) |
| Republican | 0.006 | -0.124 | -0.005 | -0.094 | -0.134 | -0.246 | -0.206 | -0.203 |
|  | (0.967) | (0.359) | (0.973) | (0.478) | (0.339) | (0.062) | (0.212) | (0.198) |
| Ideology | -0.183* |  | -0.125 |  | -0.148* |  | 0.004 |  |
|  | (0.020) |  | (0.106) |  | (0.030) |  | (0.962) |  |
| Religion | -0.086 | -0.106* | -0.095* | -0.108** | -0.143*** | -0.159*** | -0.104* | -0.102* |
|  | (0.053) | (0.014) | (0.024) | (0.009) | (0.000) | (0.000) | (0.021) | (0.020) |
| Female | 0.065 | 0.086 | 0.170 | 0.185 | -0.080 | -0.064 | -0.224 | -0.223 |
|  | (0.584) | (0.473) | (0.142) | (0.109) | (0.448) | (0.546) | (0.050) | (0.050) |
| Uninsured | -0.660* | -0.602* | -0.570* | -0.542* | -0.880*** | -0.851** | -0.943* | -0.943* |
|  | (0.011) | (0.022) | (0.025) | (0.036) | (0.001) | (0.001) | (0.014) | (0.014) |
| Medicaid | -0.487* | -0.467* | -0.549** | -0.535** | -0.707*** | -0.698*** | -0.646** | -0.644** |
|  | (0.013) | (0.018) | (0.003) | (0.004) | (0.000) | (0.000) | (0.002) | (0.002) |
| Employer-sponsored insurance | 0.372* | 0.388* | 0.268 | 0.280 | 0.125 | 0.145 | -0.138 | -0.139 |
|  | (0.040) | (0.033) | (0.116) | (0.101) | (0.428) | (0.354) | (0.427) | (0.422) |
| Medicare | -0.070 | -0.057 | -0.149 | -0.142 | -0.111 | -0.098 | -0.229 | -0.231 |
|  | (0.722) | (0.772) | (0.438) | (0.463) | (0.531) | (0.581) | (0.227) | (0.223) |
| Non-Hispanic White | -0.188 | -0.182 | 0.165 | 0.169 | -0.003 | 0.010 | -0.476 | -0.477 |
|  | (0.528) | (0.540) | (0.560) | (0.550) | (0.991) | (0.972) | (0.085) | (0.085) |
| Non-Hispanic Black | -0.338 | -0.339 | 0.021 | 0.018 | -0.471 | -0.471 | -1.214*** | -1.214*** |
|  | (0.315) | (0.312) | (0.947) | (0.956) | (0.166) | (0.165) | (0.000) | (0.000) |
| Non-Hispanic Asian | 1.003 | 1.015 | 1.319* | 1.327* | 0.742 | 0.748 | -0.234 | -0.233 |
|  | (0.097) | (0.090) | (0.014) | (0.013) | (0.060) | (0.059) | (0.511) | (0.512) |
| Hispanic | -0.278 | -0.269 | 0.027 | 0.028 | -0.243 | -0.237 | -0.799* | -0.798* |
|  | (0.409) | (0.424) | (0.932) | (0.932) | (0.465) | (0.476) | (0.016) | (0.016) |
| Income | 0.130*** | 0.126** | 0.133*** | 0.131*** | 0.052 | 0.050 | 0.049 | 0.050 |
|  | (0.001) | (0.001) | (0.000) | (0.001) | (0.152) | (0.162) | (0.203) | (0.200) |
| Education | 0.286*** | 0.293*** | 0.264*** | 0.268*** | 0.273*** | 0.275*** | 0.211** | 0.211** |
|  | (0.000) | (0.000) | (0.000) | (0.000) | (0.000) | (0.000) | (0.003) | (0.003) |
| Age | -0.041 | -0.042 | -0.045* | -0.045* | -0.050** | -0.051** | -0.027 | -0.027 |
|  | (0.062) | (0.059) | (0.036) | (0.034) | (0.007) | (0.006) | (0.187) | (0.187) |
| Age^2^ | 0.001** | 0.001** | 0.001*** | 0.001*** | 0.001*** | 0.001*** | 0.001** | 0.001** |
|  | (0.004) | (0.005) | (0.001) | (0.001) | (0.000) | (0.000) | (0.007) | (0.007) |
| Constant | -4.302*** | -4.925*** | -4.824*** | -5.264*** | -4.988*** | -5.479*** | -7.478*** | -7.472*** |
|  | (0.000) | (0.000) | (0.000) | (0.000) | (0.000) | (0.000) | (0.000) | (0.000) |
|  |  |  |  |  |  |  |  |  |
| Observations | 3,774 | 3,782 | 3,774 | 3,782 | 3,774 | 3,782 | 3,774 | 3,782 |

Notes: All models presented are logit models estimated with survey weights. p-values in parentheses. AME is average marginal effect. *** p<0.001, ** p<0.01, * p<0.05.

Source: Authors’ survey

**Appendix 10:** Respondents’ Intention to Vaccinate in the Future

Source: Authors’ survey.

Notes: Confidence intervals omitted for clarity.

**Appendix 11:** Correlates of Future Vaccine Hesitancy for Existing Vaccine, Existing Booster, Future Vaccine, Alternative Specification

|  | (1) | (2) | (3) | (4) | (5) | (6) |
| --- | --- | --- | --- | --- | --- | --- |
| VARIABLES | **Future Vaccination with Existing Vaccine** | | **Future Vaccination with Existing Booster** | | **Future Vaccination with New Vaccine** | |
|  |  |  |  |  |  |  |
| Had COVID | -0.187** | -0.185** | -0.077 | -0.079 | -0.129** | -0.126** |
|  | (0.008) | (0.010) | (0.215) | (0.210) | (0.004) | (0.005) |
| Concerned about COVID | 0.294*** | 0.300*** | 0.312*** | 0.323*** | 0.289*** | 0.304*** |
|  | (0.000) | (0.000) | (0.000) | (0.000) | (0.000) | (0.000) |
| Perceived COVID risk | 0.013 | 0.010 | 0.065* | 0.065* | 0.063** | 0.063** |
|  | (0.692) | (0.759) | (0.037) | (0.036) | (0.003) | (0.003) |
| Tested for COVID | 0.059 | 0.057 | 0.140 | 0.144 | 0.029 | 0.032 |
|  | (0.370) | (0.396) | (0.105) | (0.100) | (0.585) | (0.553) |
| Concerned about COVID vaccine effectiveness | -0.052 | -0.056* | 0.029 | 0.026 | -0.036 | -0.039 |
|  | (0.052) | (0.040) | (0.448) | (0.499) | (0.118) | (0.093) |
| Lost someone to COVID | -0.072 | -0.068 | 0.041 | 0.039 | 0.034 | 0.031 |
|  | (0.401) | (0.436) | (0.542) | (0.553) | (0.490) | (0.524) |
| First Dose |  |  | -0.217*** | -0.208*** | -0.118* | -0.112* |
|  |  |  | (0.000) | (0.000) | (0.035) | (0.047) |
| Initial Sequence |  |  | -0.154* | -0.158** | 0.337*** | 0.335*** |
|  |  |  | (0.010) | (0.008) | (0.000) | (0.000) |
| First Booster |  |  |  |  | 0.409*** | 0.420*** |
|  |  |  |  |  | (0.000) | (0.000) |
| Second Booster |  |  |  |  | 1.037*** | 1.035*** |
|  |  |  |  |  | (0.000) | (0.000) |
| Trust in Health Institutions | 0.121** | 0.140** | 0.322*** | 0.335*** | 0.251*** | 0.267*** |
|  | (0.004) | (0.001) | (0.000) | (0.000) | (0.000) | (0.000) |
| Vaccines are safe | 0.081 | 0.080 | 0.423*** | 0.430*** | 0.225*** | 0.230*** |
|  | (0.143) | (0.152) | (0.000) | (0.000) | (0.000) | (0.000) |
| Vaccines are effective | 0.011 | 0.026 | 0.119 | 0.114 | 0.107* | 0.107* |
|  | (0.877) | (0.708) | (0.151) | (0.168) | (0.040) | (0.043) |
| Vaccines are important | 0.072 | 0.073 | 0.076 | 0.089 | 0.101* | 0.109* |
|  | (0.264) | (0.265) | (0.305) | (0.237) | (0.041) | (0.030) |
| Democrat | 0.119 | 0.164 | 0.205** | 0.272*** | 0.174** | 0.250*** |
|  | (0.308) | (0.165) | (0.008) | (0.000) | (0.001) | (0.000) |
| Republican | -0.059 | -0.128* | -0.245** | -0.346*** | -0.125* | -0.233*** |
|  | (0.395) | (0.041) | (0.002) | (0.000) | (0.025) | (0.000) |
| Ideology | -0.101* |  | -0.131*** |  | -0.143*** |  |
|  | (0.014) |  | (0.000) |  | (0.000) |  |
| Religiosity | 0.006 | -0.001 | -0.020 | -0.035 | 0.006 | -0.011 |
|  | (0.771) | (0.956) | (0.366) | (0.108) | (0.711) | (0.480) |
| Female | -0.090 | -0.073 | -0.077 | -0.074 | -0.016 | -0.001 |
|  | (0.149) | (0.241) | (0.199) | (0.219) | (0.694) | (0.984) |
| Uninsured | -0.060 | -0.039 | -0.149 | -0.120 | -0.083 | -0.051 |
|  | (0.598) | (0.738) | (0.324) | (0.426) | (0.376) | (0.590) |
| Medicaid | -0.112 | -0.105 | -0.028 | -0.018 | -0.035 | -0.022 |
|  | (0.272) | (0.300) | (0.788) | (0.862) | (0.640) | (0.768) |
| Employer-sponsored insurance | -0.158 | -0.150 | -0.136 | -0.124 | -0.062 | -0.044 |
|  | (0.064) | (0.081) | (0.121) | (0.154) | (0.306) | (0.469) |
| Medicare | 0.046 | 0.052 | 0.065 | 0.075 | -0.015 | -0.003 |
|  | (0.670) | (0.633) | (0.524) | (0.462) | (0.834) | (0.971) |
| Non-Hispanic White | -0.116 | -0.122 | -0.178 | -0.167 | -0.098 | -0.094 |
|  | (0.460) | (0.441) | (0.275) | (0.319) | (0.340) | (0.364) |
| Non-Hispanic Black | -0.136 | -0.153 | -0.151 | -0.139 | -0.209 | -0.209 |
|  | (0.490) | (0.439) | (0.419) | (0.466) | (0.096) | (0.098) |
| Non-Hispanic Asian | 0.185 | 0.166 | 0.077 | 0.093 | 0.063 | 0.059 |
|  | (0.504) | (0.553) | (0.696) | (0.641) | (0.629) | (0.648) |
| Hispanic | -0.014 | -0.027 | -0.067 | -0.059 | -0.055 | -0.055 |
|  | (0.941) | (0.883) | (0.716) | (0.755) | (0.658) | (0.660) |
| Income | 0.032 | 0.027 | -0.019 | -0.020 | 0.003 | 0.000 |
|  | (0.199) | (0.279) | (0.324) | (0.296) | (0.843) | (0.974) |
| Education | -0.022 | -0.013 | 0.062 | 0.062 | 0.055* | 0.058* |
|  | (0.585) | (0.757) | (0.090) | (0.092) | (0.041) | (0.032) |
| Age | -0.046*** | -0.046*** | 0.013 | 0.012 | -0.007 | -0.008 |
|  | (0.000) | (0.000) | (0.172) | (0.208) | (0.336) | (0.278) |
| Age^2^ | 0.000** | 0.000** | -0.000 | -0.000 | 0.000 | 0.000 |
|  | (0.004) | (0.004) | (0.462) | (0.462) | (0.163) | (0.175) |
| Constant | 2.118*** | 1.754*** | -0.757 | -1.174** | -0.044 | -0.519* |
|  | (0.000) | (0.000) | (0.058) | (0.002) | (0.863) | (0.033) |
|  |  |  |  |  |  |  |
| Observations | 1,057 | 1,060 | 2,203 | 2,207 | 3,743 | 3,750 |
| R-squared | 0.322 | 0.314 | 0.422 | 0.416 | 0.611 | 0.606 |

Notes: All models presented are OLS models estimated with survey weights. p-values in parentheses. *** p<0.001, ** p<0.01, * p<0.05.

Source: Authors’ survey

**Appendix 12**: Support for Federal Funding for COVID-19 Test, Vaccines, and Treatments

Source: Authors’ survey.

Notes: Confidence intervals omitted for clarity.

**Appendix 13:** Support for Federal Funding for COVID-19 Tests, Vaccines, and Treatments, Alternative Specification

|  | (1) | (2) | (3) | (4) | (5) | (6) |
| --- | --- | --- | --- | --- | --- | --- |
| VARIABLES | **Pay for Tests** | | **Pay for Vaccines** | | **Pay for Treatments** | |
|  |  |  |  |  |  |  |
| Had COVID | 0.036 | 0.043 | 0.018 | 0.024 | 0.003 | 0.010 |
|  | (0.458) | (0.379) | (0.706) | (0.617) | (0.952) | (0.848) |
| Concerned about COVID | 0.108*** | 0.121*** | 0.118*** | 0.131*** | 0.110*** | 0.122*** |
|  | (0.000) | (0.000) | (0.000) | (0.000) | (0.000) | (0.000) |
| Perceived COVID risk | -0.022 | -0.024 | -0.022 | -0.024 | 0.020 | 0.018 |
|  | (0.319) | (0.295) | (0.329) | (0.291) | (0.405) | (0.463) |
| Tested for COVID | 0.177** | 0.185** | 0.073 | 0.080 | 0.102 | 0.111 |
|  | (0.005) | (0.003) | (0.249) | (0.208) | (0.121) | (0.093) |
| Concerned about COVID vaccine effectiveness | 0.106*** | 0.101*** | 0.088** | 0.082** | 0.111*** | 0.106*** |
|  | (0.000) | (0.000) | (0.001) | (0.003) | (0.000) | (0.000) |
| Lost someone to COVID | 0.134** | 0.137** | 0.092* | 0.096* | 0.115* | 0.118* |
|  | (0.004) | (0.004) | (0.045) | (0.038) | (0.024) | (0.021) |
| First Dose | 0.019 | 0.025 | 0.068 | 0.070 | 0.067 | 0.072 |
|  | (0.713) | (0.636) | (0.178) | (0.165) | (0.232) | (0.197) |
| Initial Sequence | 0.117* | 0.116* | 0.149** | 0.149** | 0.054 | 0.054 |
|  | (0.017) | (0.018) | (0.002) | (0.002) | (0.291) | (0.301) |
| First Booster | 0.007 | 0.015 | 0.016 | 0.027 | -0.075 | -0.068 |
|  | (0.899) | (0.773) | (0.746) | (0.579) | (0.193) | (0.242) |
| Second Booster | 0.033 | 0.027 | 0.143** | 0.137** | 0.102 | 0.096 |
|  | (0.533) | (0.604) | (0.003) | (0.005) | (0.074) | (0.095) |
| Trust in Health Institutions | 0.209*** | 0.231*** | 0.228*** | 0.253*** | 0.121** | 0.143*** |
|  | (0.000) | (0.000) | (0.000) | (0.000) | (0.001) | (0.000) |
| Vaccines are safe | -0.041 | -0.035 | -0.050 | -0.043 | -0.053 | -0.047 |
|  | (0.453) | (0.534) | (0.369) | (0.446) | (0.351) | (0.417) |
| Vaccines are effective | 0.055 | 0.063 | 0.093 | 0.101 | 0.023 | 0.031 |
|  | (0.350) | (0.292) | (0.133) | (0.105) | (0.720) | (0.633) |
| Vaccines are important | 0.149** | 0.156** | 0.178** | 0.187*** | 0.149** | 0.156** |
|  | (0.006) | (0.005) | (0.001) | (0.001) | (0.007) | (0.005) |
| Democrat | 0.078 | 0.157** | 0.065 | 0.153** | 0.070 | 0.146** |
|  | (0.117) | (0.001) | (0.188) | (0.002) | (0.222) | (0.009) |
| Republican | 0.020 | -0.084 | 0.111 | -0.003 | 0.068 | -0.031 |
|  | (0.745) | (0.176) | (0.066) | (0.965) | (0.288) | (0.622) |
| Ideology | -0.145*** |  | -0.159*** |  | -0.137*** |  |
|  | (0.000) |  | (0.000) |  | (0.000) |  |
| Religiosity | -0.025 | -0.042* | -0.052** | -0.069*** | -0.031 | -0.047** |
|  | (0.136) | (0.010) | (0.002) | (0.000) | (0.077) | (0.006) |
| Female | 0.222*** | 0.236*** | 0.238*** | 0.256*** | 0.204*** | 0.217*** |
|  | (0.000) | (0.000) | (0.000) | (0.000) | (0.000) | (0.000) |
| Uninsured | -0.076 | -0.037 | -0.083 | -0.052 | -0.082 | -0.045 |
|  | (0.488) | (0.740) | (0.406) | (0.612) | (0.442) | (0.674) |
| Medicaid | 0.083 | 0.084 | -0.016 | -0.011 | 0.148 | 0.148 |
|  | (0.265) | (0.267) | (0.838) | (0.883) | (0.058) | (0.064) |
| Employer-sponsored insurance | -0.021 | -0.005 | -0.025 | -0.008 | -0.043 | -0.028 |
|  | (0.759) | (0.941) | (0.716) | (0.910) | (0.556) | (0.704) |
| Medicare | 0.085 | 0.092 | 0.037 | 0.045 | 0.072 | 0.079 |
|  | (0.234) | (0.200) | (0.603) | (0.526) | (0.359) | (0.315) |
| Non-Hispanic White | -0.078 | -0.079 | -0.151 | -0.152 | -0.203* | -0.205* |
|  | (0.408) | (0.409) | (0.084) | (0.088) | (0.025) | (0.025) |
| Non-Hispanic Black | 0.068 | 0.043 | -0.024 | -0.048 | -0.055 | -0.081 |
|  | (0.534) | (0.701) | (0.816) | (0.660) | (0.616) | (0.472) |
| Non-Hispanic Asian | -0.045 | -0.052 | 0.030 | 0.022 | -0.110 | -0.117 |
|  | (0.728) | (0.691) | (0.800) | (0.859) | (0.395) | (0.365) |
| Hispanic | -0.008 | -0.012 | -0.036 | -0.044 | -0.116 | -0.120 |
|  | (0.941) | (0.917) | (0.731) | (0.677) | (0.278) | (0.267) |
| Income | -0.042** | -0.045** | -0.050*** | -0.053*** | -0.025 | -0.029 |
|  | (0.002) | (0.001) | (0.000) | (0.000) | (0.091) | (0.054) |
| Education | -0.143*** | -0.136*** | -0.107*** | -0.101*** | -0.119*** | -0.112*** |
|  | (0.000) | (0.000) | (0.000) | (0.000) | (0.000) | (0.000) |
| Age | 0.016* | 0.014* | 0.020** | 0.019** | 0.024** | 0.023** |
|  | (0.028) | (0.046) | (0.003) | (0.006) | (0.002) | (0.004) |
| Age^2^ | -0.000 | -0.000 | -0.000* | -0.000* | -0.000** | -0.000* |
|  | (0.078) | (0.084) | (0.011) | (0.011) | (0.009) | (0.011) |
| Constant | 2.915*** | 2.414*** | 2.786*** | 2.233*** | 2.838*** | 2.362*** |
|  | (0.000) | (0.000) | (0.000) | (0.000) | (0.000) | (0.000) |
|  |  |  |  |  |  |  |
| Observations | 3,769 | 3,777 | 3,772 | 3,780 | 3,772 | 3,780 |
| R-squared | 0.223 | 0.213 | 0.257 | 0.246 | 0.161 | 0.152 |

Notes: All models presented are OLS models estimated with survey weights. p-values in parentheses. *** p<0.001, ** p<0.01, * p<0.05.

Source: Authors’ survey
